# Complexities to consider when communicating risk of COVID-19

**DOI:** 10.1016/j.puhe.2020.07.015

**Published:** 2020-09

**Authors:** M. Skovdal, M. Pickles, T.B. Hallett, C. Nyamukapa, S. Gregson

**Affiliations:** Department of Public Health, University of Copenhagen, Øster Farimagsgade 5B, 1014 Copenhagen, Denmark; School of Public Health, Imperial College London, St Mary's Hospital, London, W2 1PG, UK; School of Public Health, Imperial College London, St Mary's Hospital, London, W2 1PG, UK; Manicaland Centre for Public Health Research, Biomedical Research and Training Institute, 10 Seagrave Road, Avondale, Harare, Zimbabwe

The response to the spread of severe acute respiratory syndrome coronavirus 2 (SARS-CoV-2) around the world has so far been characterised by governments issuing instructions about the action to take. However, as governments begin to ease restrictions, the potential for coronavirus disease 2019 (COVID-19) to spread is increased. We argue that correct understanding of individuals' risks of becoming infected and dying is a prerequisite for people and communities to take responsibility and engage in prevention practices, both for self and others, and also to reduce unnecessary anxieties and other unintended negative outcomes. At the same time, effective communication of these risks is fraught with difficulty and there are important complexities and social constraints that must be recognised and addressed. In our view, there has been little scientific discussion on the complexities, social determinants and impacts of COVID-19 risk communication. Here, we highlight seven major complexities in communicating risk and suggest directions for addressing these (Table1). They serve as a framework for governments, researchers, policy and public health workers to critically appraise COVID-19 risk messaging efforts. As we are trying to highlight complexities that are widely applicable (rather than specific to certain countries or regions), their relevance will differ from context to context.

**Table 1:** Considerations and recommendations to communicate risk in the COVID-19 response.

**Communicate variation in risk** • Avoid oversimplified ‘one-size-fits-all’ risk messages • Distinguish between risk of SARS-CoV-2 infection and risk of severe COVID-19 disease • Target risk messages to people according to their levels of risk and capacity to adopt alternative prevention methods • Communicate the uncertainty of risk estimates and that new data may lead to changes **Protect against unintended outcomes of COVID-19 prevention measures** • Develop risk messaging that reflects the broader socio-economic and health context and is actionable by local people. • Include messaging to mitigate other forms of risk (e.g. young women should still adhere to government advice but not put off trips to hospital for breast cancer screening) **Avoid negative social consequences of risk messaging** • Avoid using unhelpful metaphors (e.g. war, enemy) in risk messaging. • Avoid using language that can cast shame or blame to people **Tackle misinformation** • Monitor the emergence and spread of myths and misinformation on social media and within the community • Use locally trusted institutions and individuals to address misinformation and channels that are widely used by the relevant population • Promote trust in official sources by ensuring that messaging from all such sources is consistent **Reflect changes in the nature of risk and risk perception as the epidemic evolves** • Review, revise and explain changes in risk messages as the epidemic evolves • Develop risk messages that counteract innate tendencies for message ‘fatigue’ **Promote motivation and creative capacity** • Use data on risk to stimulate and strengthen motivation to follow government guidance • Encourage people to think creatively and tailor prevention methods to their own circumstances (e.g. to find effective ways to shield vulnerable family members) • Foster a sense of collective responsibility (e.g. risk messaging that emphasises that your actions benefit others) **Consider the broader social determinants of risk** • Recognise and address social and health inequities, social norms, discrimination and political agendas, which put some people at greater risk or prevent them from engaging with risk-reducing practices. • Make freely available health services and equipment to assist risk-reducing practices

## Seven challenges and recommendations for communicating risk

One: The risks of acquiring SARS-CoV-2 infection and of dying from COVID-19 disease once infected vary considerably by epidemic context and between individuals.^1^ Nevertheless, it is apparent that the risk of infection varies with the stage of the epidemic, which varies by microregion, and an individual's exposure, which is often much higher for healthcare workers and carers and elevated for those with jobs that cannot be carried out from home, amongst whom ethnic minority groups and people living in greater deprivation may be overrepresented.^2^ The risk of death from COVID-19, given infection, varies substantially according to age, male sex, obesity and other factors.^3^ Thus, there is no ‘one number’ to quote to people for their risk; but, at the same time, everyone should know the range in which their risk is likely to fall. Finding ways to provide clear and targeted information about who is at increased risk whilst also recognising the intersectionality of these factors is essential.

Two: Unintended outcomes – such as anxiety, avoiding going to work and limited healthcare seeking – can result for some people. Thus, overestimating one's own risk could be as unhelpful to economic well-being and health overall as understating one's own risk. Moreover, some people aware of their individual risk may (un)willingly take risks, for instance, by making a trade-off between risk and maintaining a livelihood. Communicating risk of SARS-CoV-2 infection must be considered in the broader context of a group of risks as great or greater than that from COVID-19.^4^ Therefore, developing strategies to mitigate these risks is important too.^4^

Three: How we communicate risks can have negative wider social consequences. Messages about the ‘new risk’ may reproduce constructions of COVID-19 as a ‘foreign invader’, facilitating stigma and xenophobia.^5^ Risk communication may also construct new social norms about how to act and behave in public, which, inadvertently, contribute to blaming and shaming those who are unable to comply – disproportionately affecting already stigmatised groups.

Four: In our view, there has been little communication of actual risk to the individual about the risk involved, and into this vacuum, misinformation and misunderstanding have proliferated. When risks of acquiring SARS-CoV-2 infection and of dying from COVID-19 then are communicated, it takes place in an arena with a lot of background noise, including misinformation that is sometimes deliberate,^6^ and distrust of medical information. Communication about risk needs to cut through this noise by working with the different channels of communication (social media, community groups, local leadership structures, public campaigns) that people listen to and creating community knowledge and trust in public bodies that act to prevent amplification of misinformation.

Five: Self-perception of risk is not static but evolves constantly with the epidemic for the right reason (risk of infection is genuinely dynamic in the course of an epidemic) and the wrong reason (persons can acclimatize to a risk and risk compensation can set in). When an epidemic starts, risk communication messaging arguably needs to be harder hitting than later when people already feel ‘at-risk’ and taking preventative steps has become the ‘new normal’. Over time and as evidence and messaging gets updated, fear of infection, which can be a key predictor of risk-reducing behaviour change, may be replaced by ambiguities, individualistic perspectives on the response, personal experiences and values as key determinants.^7^ Adapting risk messaging to the epidemiology of COVID-19 will be critical to maintain positive behaviour change.

Six: Risk involves both risk to self and risk to the community, and prevention measures may protect the individual (e.g. hand washing), close contacts and the wider community (e.g. face covering), or both (social distancing). Improving people's accurate risk perception – and an understanding of how their own behaviour affects the risks of others – is essential to strengthen their resolve in reducing transmission and their capacity to creatively find ways to shield themselves and others from infection. Even when substantial pharmaceutical interventions become available, their uptake may be affected by similar considerations, with computation of one's own risk being further complicated by factors including the effectiveness and local coverage of vaccines.

Finally, Seven: Improving risk perception in isolation from broader social determinants and impacts of risk are unlikely to result in an effective communication strategy. COVID-19 is affecting disproportionately certain strata of society, with some population groups at a disadvantage with regards to access to services, housing, employment and so on. Risk communication cannot ignore these determinants. Individuals may have sound understandings of risks of becoming infected and dying and are yet unable to engage or comply with public health messages (e.g. wear a mask if you do not have any; get tested if you have no access; access treatment if you will have to bear the cost of treatment; stay home if you are asymptomatic SARS-CoV-2-positive; respect distancing if you live in a crowded household and so on).

## Going forward

Theory and prior experience strongly suggest that individuals' understanding of their own risk of infection and death from COVID-19 is crucial for adopting new behaviours that are tailored for their own risk, in addition to helping motivate adoption of generalised public health messaging.^8^ This is not easy – the risk varies over time and between persons in ways that most societies are only beginning to understand – and messaging must be considered in the context of many complexities, which are often rooted in social and health inequities, social norms and discrimination, political agendas and other features of our society. Nevertheless, this is a crucial endeavour – every bit as useful as the construction of the generalised policy directives – and attention must be increasingly devoted to it.

## Author statements

### Funding

The authors are supported by the 10.13039/100000025National Institute of Mental Health (www.nimh.nih.gov) (Grant R01MH114562–01) and the 10.13039/100000865Bill and Melinda Gates Foundation (www.gatesfoundation.org) (OPP1161471). MRP, TBH, CAN and SG acknowledge joint MRC Centre for Global Infectious Disease Analysis funding from the UK Medical Research Council and Department for International Development (grant number MR/R015600/1). The content is solely the responsibility of the authors and does not necessarily represent the official views of the NIMH or the BMGF. The funders had no role in the conception or preparation of the manuscript.

### Competing interests

The authors declare no potential conflict of interest in the commentary.
